# Efficacy of 3% hydrogen peroxide solution in cleaning tongue coating before and after surgery: a randomized phase II study

**DOI:** 10.1186/s12903-022-02325-9

**Published:** 2022-07-15

**Authors:** Sakiko Soutome, Mitsunobu Otsuru, Saki Hayashida, Tomofumi Naruse, Kota Morishita, Kazumi Kurihara, Yumiko Kawashita, Madoka Funahara, Masahiro Umeda, Hideki Taniguchi, Toshiyuki Saito

**Affiliations:** 1 Department of Oral Health, Nagasaki University Graduate School of Biomedical Science, 1-7-1 Sakamoto, Nagasaki, 852-8588 Japan; 2 Department of Clinical Oral Oncology, Nagasaki University Graduate School of Biomedical Sciences, Nagasaki, Japan; 3 Department of Dentistry and Oral Surgery, Japanese Red Cross Nagasaki Genbaku Hospital, Nagasaki, Japan; 4 School of Oral Health Sciences, Kyushu Dental University, Fukuoka, Japan; 5 Department of Breast and Endocrine Surgery, Japanese Red Cross Nagasaki Genbaku Hospital, Nagasaki, Japan

**Keywords:** Tongue coating, Number of bacteria, Hydrogen peroxide, Tongue cleaning, Cancer surgery

## Abstract

**Background:**

Increased bacterial presence in the tongue coating and thereby, the saliva, may be a risk factor for postoperative complications such as surgical site infection or postoperative pneumonia after cancer surgery. However, no method for cleaning tongue coating has been established experimentally. The purpose of this study was to verify the effect of brushing with 3% hydrogen peroxide on suppression of the number of bacteria in tongue coating.

**Methods:**

Sixteen patients with gastric cancer or colorectal cancer undergoing surgery were randomly allocated to control and intervention groups. In the control group, the tongue was brushed for 30 s with a water-moistened toothbrush, while in the intervention group, the tongue was brushed for 30 s with a toothbrush moistened with 3% hydrogen peroxide. Bacterial counts on tongue coating were measured before and 30 s after cleaning the tongue coating using the Rapid Oral Bacteria Quantification System.

**Results:**

In the control group, the number of bacteria on the tongue did not decrease significantly after tongue cleaning on the day before surgery, but did on the day after surgery. In contrast, in the intervention group, the number of bacteria on the tongue decreased significantly after tongue cleaning both on the day before and the day after surgery. Furthermore, when comparing the control and intervention groups, the intervention group had a greater reduction effect.

**Conclusions:**

Tongue brushing with 3% hydrogen peroxide is a useful method to reduce the number of bacteria on the tongue in patients with gastrointestinal cancer undergoing surgery.

*Trial registration* jRCTs071200020 (July 3, 2020).

## Background

Tongue coating is a white moss-like deposit that covers the surface of the mucosa on the dorsum of the tongue and is usually physiologically present and not pathological. However, excessive tongue coating is known to be a major cause of bad breath [1]; furthermore, it may cause diseases such as aspiration pneumonia in older adults [2, 3]. Aspiration pneumonia develops when three factors—pathogenic microorganisms in the saliva, dysphagia, and weakened immunity—are present simultaneously. In elderly or post-surgery patients, weakened immunity and dysphagia are often unavoidable; therefore, reducing the salivary bacterial counts is necessary to prevent the occurrence of aspiration pneumonia.

Previously, we investigated the relationship between various clinical factors and the number of salivary bacteria in 120 elderly people requiring care and found that tube feeding, inability to gargle, and a higher number of bacteria on the tongue coating were independent risk factors for bacterial growth in the saliva [4]. Furthermore, our prospective observational study of 54 patients who underwent oncologic or cardiac surgery under general anesthesia revealed that increased amount of tongue coating after surgery was associated with increased bacterial count in the saliva, which suggested that tongue coating should be removed after surgery to minimize the risk of postoperative aspiration pneumonia in patients undergoing major oncologic or cardiac surgery [5].

However, although there are various reports on cleaning tongue coating, none have been scientifically established. We previously reported in a randomized controlled trial using 32 adult participants that tongue brushing with 10% povidone iodine or 3% hydrogen peroxide (OX) was the most effective method for reducing the bacterial count in tongue coating [6]. The purpose of this study was to clarify whether the number of bacteria coating the tongue was reduced by cleaning the tongue with OX solution on the day before and the day after surgery in patients undergoing gastrointestinal cancer surgery.

## Preliminary research

**Fig. 1:**
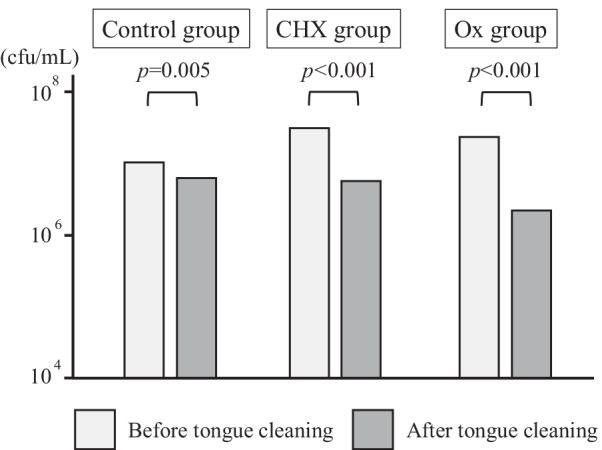
The number of bacteria on the tongue before and after tongue cleaning with water (control group), chlorhexidine (CHX group), and hydrogen peroxide (OX group). Bacterial count decreased in each group, but OX group showed the largest reduction effect

As a preliminary experiment, we compared the effects of chlorhexidine (CHX) and OX solutions on reducing bacterial numbers on the tongue at concentrations approved for oral use in Japan. This study was approved by the Institutional Review Board of Nagasaki University, and consent for participation and publication was obtained by each participant. Twenty-four subjects who visited Nagasaki University Hospital Dentistry for dental maintenance were allocated into three groups: (1) control group, (2) CHX group, and (3) OX group. In the control, CHX, and OX groups, the tongue was brushed for 30 s with water, CHX (0.005% chlorhexidine gluconate, ConCool F®, Weltec Co., Ltd., Osaka, Japan), and OX (3% OX solution, Oxydol®, Kenei Pharmaceutical Co., Ltd.)-moistened toothbrushes, respectively, and gargled with water. We found that the number of bacteria on the tongue was significantly reduced in both the CHX and OX groups as compared with the control group, but the OX group had the largest reduction effect (Fig. 1). Therefore, we used 3% OX solution in this study to investigate its effect on reducing the number of bacteria in tongue coating in patients with gastrointestinal cancer.

## Materials and methods

### Study design

This was a randomized controlled phase II study to investigate the efficacy of 3% OX solution in reducing bacterial numbers in tongue coating in patients undergoing surgery for gastric or colorectal cancer. The subjects were 16 adult patients with gastric cancer or colorectal cancer who underwent surgery at the Japanese Red Cross Nagasaki Genbaku Hospital between June 2020 and September 2021. This is a preliminary study, so, we set the number of cases to a small number of 16 cases. Those without macroscopic tongue coating were excluded from the study. Patients were referred to in-hospital dentistry when their surgeries were scheduled and received oral examinations including panoramic X-ray, oral hygiene instructions, removal of dental plaque and calculus, and dental treatment including extraction the tooth that was difficult to preserve. These dental treatments were completed at least one week before surgery. They underwent mechanical tooth cleaning by a dental hygienist (KK) and tongue coating cleaning by a dentist (SS) the day before surgery. They were randomly allocated to control and intervention groups in a 1:1 ratio using a computer software with a simple randomization method, where, the tongue was brushed for 30 s with a water and 3% OX solution (Oxydol®, Kenei Pharmaceutical Co., Ltd.)-moistened toothbrush, respectively. One of the researchers supervised the randomization procedure. The head of the toothbrush used for cleaning the tongue was 20 mm × 9 mm in size. Further, it was a flat toothbrush with medium bristle hardness and a straight grip. This was followed by patients gargling with water in both groups. After oral care by a dentist and a dental hygienist the day before surgery, no oral care intervention by a dentist, dental hygienist, nurse, etc. was performed until the second measurement of this study was performed the day after surgery. Tongue cleaning and the measurement was performed from 10 to 12 AM by a single dentist (SS). All patients had not yet been orally fed at the time of measurement.

### Measurement of bacterial count on tongue coating

Bacterial counts on tongue coating were measured before and 30 s after cleaning the tongue coating and gargling, using the Rapid Oral Bacteria Quantification System (DU-AA01NP-H Panasonic Healthcare Co. Ltd., Osaka, Japan), which is based on dielectrophoresis and impedance measurements [7, 8]. To collect a sample, a sterile cotton swab was placed on the attached constant-pressure sample collection device, pressed against the center of the dorsum of the tongue, and rubbed back and forth for a length of approximately 2 cm.

### Statistical analysis

Endpoint was the total number of bacteria in the tongue coating before and after tongue cleaning on the day before and after surgery for gastrointestinal cancer. The differences in bacterial counts before and after surgery, and between the control and intervention groups were analyzed using paired and unpaired t-tests, respectively. All statistical analyses were performed using SPSS software (version 26.0; Japan IBM Co., Ltd., Tokyo, Japan). Statistical significance was set at p < 0.05.

## Results

### Patient characteristics

The characteristics of patients in the control and intervention groups are shown in Table 1. There were 13 men and 3 women in the group, with an average age of 68.2 years. There was no significant difference in background factors between the two groups, except that there were more patients with low albumin levels in the intervention group. There were no adverse events following tongue cleaning by OX, and no postoperative pneumonia in 16 patients in the study.

**Table 1:** Patient characteristics

Variable	Control group	Intervention group	*p* value
Sex			
Male	7	6	1.000
Female	1	2	
Age (years)	67.9 ± 11.4	68.5 ± 7.84	0.900
BMI	22.5 ± 1.92	23.5 ± 3.73	0.522
Primary disease			
Stomach cancer	3	2	1.000
Colon cancer	5	6	
Smoking habit			
(−)	5	5	1.000
(+)	3	3	
Drinking habit			
(−)	5	4	1.000
(+)	3	4	
Diabetes			
(−)	7	5	0.569
(+)	1	3	
Leukocyte (μL)	6425 ± 1649	7275 ± 2656	0.455
Hemoglobin (g/dL)	12.9 ± 2.20	12.6 ± 2.22	0.798
Albumin (g/dL)	4.39 ± 0.340	3.90 ± 0.323	0.011
Creatinine (mg/dL)	0.889 ± 0.0794	0.880 ± 0.180	0.902
Number of teeth	15.0 ± 13.0	18.6 ± 11.3	0.561
Denture use			
(−)	4	5	1.000
(+)	4	3	
Operation time			
Minutes	163 ± 68.8	210 ± 65.0	0.184
Oral feeding on the next day after surgery			
(−)	5	7	0.569
(+)	3	1	

### Number of bacteria on dorsum of the tongue before and after surgery

Table 2 shows the relationship between the number of bacteria on dorsum of the tongue before surgery and each variable. Univariate analysis showed a significant positive correlation between the number of teeth remaining and the number of bacteria on the tongue of preoperative patients (*p* = 0.045), but no other variables were associated with the bacterial count.

**Table 2:** Relationship between each variable and number of total bacteria on the tongue at the day before surgery

Variable		*p* value
(i) Categorical variable	Number of total bacteria (logarithm)	
Sex		
Female	7.82 ± 0.316	0.186§
Male	7.44 ± 0.430	
Primary disease		
Stomach cancer	7.41 ± 0.583	0.511§
Colon cancer	7.56 ± 0.362	
Smoking habit		
(−)	7.54 ± 0.474	0.753§
(+)	7.47 ± 0.378	
Drinking habit		
(−)	7.52 ± 0.489	0.963§
(+)	7.51 ± 0.375	
Diabetes		
(−)	7.51 ± 0.335	0.902§
(+)	7.54 ± 0.711	
Denture use		
(−)	7.69 ± 0.373	0.056§
(+)	7.29 ± 0.406	
(ii) Variable (continuous variable)	Spearman's correlation coefficient	
Age (years)	0.005	0.985^†^
Body mass index	0.037	0.892^†^
Leukocyte (μL)	0.136	0.615^†^
Hemoglobin (g/dL)	− 0.349	0.185^†^
Albumin (g/dL)	− 0.329	0.213^†^
Creatinine (mg/dL)	− 0.165	0.541^†^
Number of teeth	0.507	0.045^†^

^§^One-way ANOVA, ^†^Spearman's rank correlation coefficient

The number of bacteria on the tongue on the day after surgery was significantly higher in patients who had a high number of bacteria before and after cleaning the tongue before surgery (Table 3).

**Table 3:** Relationship between each variable and number of total bacteria on the tongue at the day after surgery

Variable		*p* value
(i) Categorical variable	Number of total bacteria (logarithm)	
Sex		
Female	7.40 ± 0.288	0.454§
Male	7.05 ± 0.753	
Primary disease		
Stomach cancer	7.36 ± 0.493	0.373§
Colon cancer	7.01 ± 0.767	
Smoking habit		
(−)	7.25 ± 0.450	0.331§
(+)	6.89 ± 0.996	
Drinking habit		
(−)	6.93 ± 0.839	0.231§
(+)	7.36 ± 0.389	
Diabetes		
(−)	7.30 ± 0.434	0.068§
(+)	6.57 ± 1.10	
Oral feeding after surgery		
(−)	7.21 ± 0.363	0.383§
(+)	6.84 ± 1.34	
Denture use		
(−)	7.19 ± 0.883	0.643§
(+)	7.02 ± 0.390	
(ii) Variable (continuous variable)	Spearman's correlation coefficient	
Age (years)	− 0.010	0.970^†^
Body mass index	0.082	0.762^†^
Leukocyte (μL)	− 0.084	0.757^†^
Hemoglobin (g/dL)	− 0.296	0.266^†^
Albumin (g/dL)	− 0.240	0.371^†^
Creatinine (mg/dL)	0.003	0.991^†^
Number of teeth	0.294	0.269^†^
Operation time (min)	− 0.278	0.297^†^
Number of baacteria before tongue cleaning at the day before surgery	0.617	0.011^†^
Number of bacteria after tongue cleaning at the day before surgery	0.697	0.003^†^

^§^One-way ANOVA, ^†^Spearman's rank correlation coefficient

### Comparison of tongue cleaning effect of the control and intervention groups

**Fig. 2:**
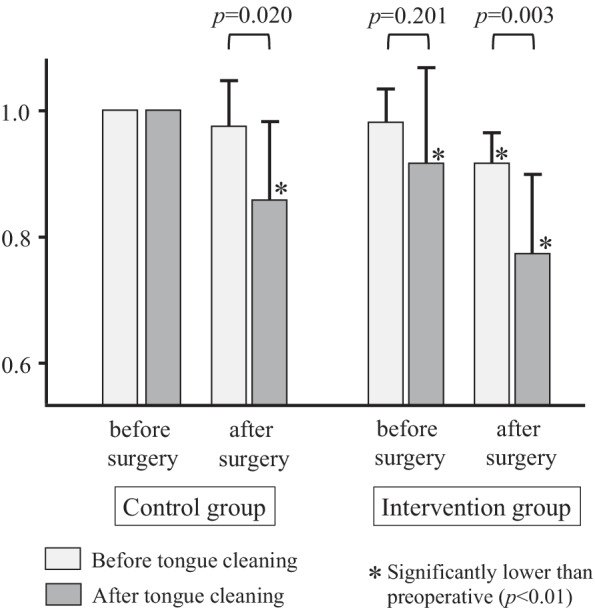
The number of bacteria on the tongue before and after tongue cleaning in the control and intervention groups. The logarithm of the number of bacteria at the baseline is 1, and it is expressed as a relative ratio. The intervention group showed a greater reduction effect than the control group

Figure 2 shows the changes in the number of bacteria on dorsum of the tongue in the control and intervention groups, with the logarithm of the number of bacteria on the day before preoperative oral care being set to 1.0. In the control group, the number of bacteria on the tongue did not decrease significantly even after tongue cleaning on the day before surgery, but it decreased after tongue cleaning on the day after surgery. In contrast, in the intervention group, the number of bacteria on the tongue decreased significantly after tongue cleaning both the day before and the day after surgery. Furthermore, when comparing the control and intervention groups, the intervention group showed a greater reduction effect.

## Discussion

This study showed that cleaning the tongue before and after surgery with OX solution can reduce the number of bacteria on the tongue in patients undergoing gastrointestinal cancer surgery.

Various complications may occur after cancer surgery. Among them, surgical site infection (SSI) after head and neck cancer or upper gastrointestinal cancer surgery, and postoperative pneumonia after esophageal cancer surgery are thought to be caused by direct exposure or aspiration of pathogenic microorganisms in saliva. Therefore, it is desirable to reduce the number of bacteria in saliva during the perioperative period as this is a controllable factor. In 2012, public medical insurance was applied for oral care before and after cancer surgery in Japan. Funahara et al. found that topical application of antimicrobial ointment on the tongue reduced the number of bacteria in saliva [9] and reported that this method can reduce the risk of SSI in oral cancer surgery with tracheotomy in a randomized controlled trial [10]. We have reported through multicenter clinical research that perioperative oral care is effective in preventing postoperative pneumonia in esophageal cancer [11–13]. As described above, various attempts have been made to reduce the number of bacteria in saliva and reduce the risk of complications by performing oral care before and after cancer surgery.

Tongue coating, along with dental plaque, may serve as a scaffold for the growth of oral bacteria and is thought to be one of the causes of aspiration pneumonia in elderly patients requiring nursing care as well as postoperative patients [2–5]. Tongue coating is formed by hyperkeratinization and elongation of the tongue papillae on the dorsal tongue, and the presence of oral bacteria, exfoliated epithelium, or food residues within the papillae. Hayashida et al. reported that tongue coating but not plaque increased the day after surgery in postoperative patients with mechanical ventilation and stated that tongue coating was more important than plaque as a risk factor for postoperative pneumonia [14]. The oral self-cleaning effect of oral feeding does not occur immediately after surgery for postoperative fasting, and tongue coating increases. Although 0.12% CHX is used worldwide for disinfection of oral bacteria, its use on mucous membranes is prohibited because of reports of anaphylaxis in Japan, and only mouthwash of 0.0001–0.0006% solution is allowed. Therefore, we previously examined effects of three types of disinfectants on removing tongue coating, namely benzethonium chloride, povidone iodine, and OX, which are permitted to be used in the oral cavity in Japan, and reported that OX solution was superior in terms of reducing the number of bacteria upon evaluating patients. In this study, we investigated the effects of OX in reducing the number of bacteria on the tongue of patients with gastrointestinal cancer undergoing surgery. It was difficult to remove the tongue coating formed before surgery easily by mechanical brushing with water, although the newly formed tongue coating after surgery could be removed relatively easily because it had not adhered firmly to the tongue yet. On the other hand, this study indicates that brushing with OX removed the tongue coating not only after surgery but also before surgery. Therefore, 3% OX solution can be used to clean the tongue before and after gastrointestinal cancer surgery.

This study has some limitations. First, it is a preliminary study using a small number of cases, and it is difficult to generalize the results obtained. Second, the endpoint is not the onset of complications, such as SSI or postoperative pneumonia, but the number of bacteria on the tongue. Subjective and objective data on oral cleanliness and tongue coating, such as OHAT and Tongue Coating Index scores were not examined in the study. Therefore, it has not been clarified whether tongue cleaning using OX actually leads to the prevention of complications. Furthermore, the total number of bacteria was investigated using the Rapid Oral Bacteria Quantification System; however, the bacterial species were not identified, and the changes in pathogenic microorganisms are unknown. A Device using the Rapid Oral Bacteria Quantification System is now covered by public health insurance system in Japan and is widely used in clinical practice in dentistry. The bacterial count measuring device used in this study is older than the current type, but the machine's performance and methods we used in this study were the same as a new type. In the future, we would like to address these issues in a larger patient sample.

## Conclusion

Tongue brushing with 3% OX is a useful method to reduce the number of bacteria present in the tongue coating in patients undergoing surgery for gastrointestinal cancer.

## Data Availability

The datasets generated during the current study are available from the corresponding author upon reasonable request.

## Abbreviations

CHX: Chlorhexidine
OX: Hydrogen peroxide
